# Effective Isolation and Characterization of Mycobacteriophages with the Ability to Lyse *Mycobacterium avium* subsp. *paratuberculosis*

**DOI:** 10.3390/v16010020

**Published:** 2023-12-22

**Authors:** Victoria K. Harman-McKenna, Jeroen De Buck

**Affiliations:** Faculty of Veterinary Medicine, University of Calgary, Calgary, AB T2N 1N4, Canada

**Keywords:** *Mycobacterium avium* subsp. *paratuberculosis*, bacteriophage therapy, phage cocktail

## Abstract

Johne’s disease (JD), a chronic infectious enteritis of ruminants, causes major economic losses in the dairy industry globally. This enteritis is caused by *Mycobacterium avium* subsp. *Paratuberculosis* (MAP). Currently there is no cure for JD and test-based culling has proved ineffective at preventing the spread. To isolate new mycobacteriophages (mbps) that can potentially be used to control JD transmission and infection on dairy farms, we optimized an isolation protocol by fecal spiking and the testing of different isolation solution compositions. Using this protocol, we successfully enhanced the yield of mbps from spiked fecal samples, elevating it from less than 1% to 59%. With this method, we isolated 14 mbps from 475 environmental samples collected from MAP-positive dairy farms, after in-sample enrichment with MAP and the fast-growing *M. smegmatis*. The sample sources included soil, manure pits, lactation barns, feces, milk, and drain water. After fingerprinting these mbps by restriction enzyme profiling, we concluded that 12 were distinct and novel. Further characterization of their host range revealed that eight were capable of lysing multiple MAP strains. We also studied the cross-resistance, lysogeny, the effect of pH and their antimycobacterial properties in milk replacer. Each novel mbp showed limited cross-resistance and prophage immunity and showed no reduction in the titer in a range of pHs after 4 h. The novel phages were also able to reduce the mycobacterial counts to zero after 8 h in milk replacer. In conclusion, these novel mbps could be considered to be used in the control strategies of JD on farms.

## 1. Introduction

*Mycobacterium avium* subsp. *paratuberculosis* (MAP) is the causative agent of a chronic infectious enteritis of ruminants known as Johne’s disease (JD) [1]. This infection can spread to youngstock through the ingestion of feed and water contaminated with infectious feces [2]. However, it is also possible for fetuses to be infected in utero and calves to be infected through infected colostrum from an infected dam [3]. JD causes significant economic losses due to decreased milk production, reduced animal welfare, and culling of infected animals [4]. In the Canadian dairy industry alone, losses are estimated at upwards of $90 million CAD per year [5]. This disease is widespread, having been reported in every country with a developed dairy industry. For these reasons, it is widely accepted that this disease is a major issue within the dairy industry on a global scale [5,6]. Moreover, MAP has been implicated as a potential zoonotic agent that could pose a risk to human health, particularly in individuals with compromised immune systems [7]. Therefore, the control of MAP infections is essential for both animal and human health. However, preventing MAP infections presents significant challenges. MAP is a hardy bacterium that can persist in the environment for long periods and is resistant to many physical and chemical agents due to its thick and hydrophobic cell wall [8]. In addition, test-based culling has proved ineffective at preventing the spread of JD due to the long incubation period of the disease [9] and limitations of current diagnostic tests to detect subclinical carriers and low-level shedders of MAP [10]. This, coupled with the lack of an effective cure, makes it challenging to control the spread of the disease.

Mycobacteriophages (mbps), viruses that can infect and kill mycobacteria, have gained much attention in recent years as potential therapeutics and alternatives to antibiotics [11]. They offer several advantages over traditional antibiotics, such as specificity and the fact that once infected by mbps, bacteria cannot survive [12]. Bacteriophages have been used for therapeutic purposes since 1919, when they were first applied to treat bacterial dysentery [13]. Subsequently, they were successfully used to combat staphylococcal infections in 1921 [14], and these early successes set the stage for the later use of phages for treating bacterial pathogens [15] and as antimicrobial agents [16] in the food industry. More recently, a review by Dedrik et al. in 2023 has highlighted the successful compassionate use of mbps that lyse *M. abscessus* in 20 patients with mycobacterial-related lung diseases [17]. Out of 20 patients treated, over 50% showed promising outcomes and a reduction in clinical signs and infection status [17]. Notably, one of these patients showed disseminated *M. avium* infection which responded well to the phage therapy [17]. Mbps also show great potential for metaphylactic use, as they present low inherent toxicity and have a single-dose potential [12,18]. However, the isolation and characterization of suitable mycobacteriophages for controlling MAP infections has proven challenging due to the difficulties of working with MAP. Not only is MAP the slowest-growing *Mycobacterium* that has been described, but it is also challenging to culture, due to the clumping of cells in the media [19]. Furthermore, Basra et al. conducted a study investigating the host range of newly isolated bacteriophages [20]. Their study found that certain phages had a limited host range, meaning that they could only infect specific strains of bacteria [20]. This has been highlighted as both an advantage and a problem for phage therapy. The main challenge of the specificity of mbps is finding phages that can effectively target the bacteria causing the infection. If a phage has a limited host range, it may not be effective against all strains of the bacteria. This can make it difficult to develop phage therapy treatments that are effective against a wide range of bacterial infections. In addition to limited host ranges, they also found some bacteria have mechanisms for resisting phage infection, such as CRISPR-Cas systems that can target and destroy phage DNA [21]. This can further limit the effectiveness of phage therapy. This study pointed out the need for further research into mycobacteriophage host range and e bacterial resistance to phages in order to develop effective phage therapy treatments. Moreover, the lack of MAP-lysing phages is a deficiency in our efforts to control MAP infections, as such isolation of new MAP lysing bacteriophages is an important step to using phages to control JD infection.

In this study, we describe the use of an optimized isolation protocol to isolate and characterize twelve mycobacteriophages, including their host range of phylogenetically distinct MAP strains, and their possible applications to control JD.

## 2. Materials and Methods

### 2.1. Bacterial Strains and Growth Media

Due to the difficulties of working with MAP, *Mycobacterium smegmatis* mc^2^155 was used as a host for phage isolation. *M. smegmatis* has long been considered a surrogate host for MAP-lysing mbps, due to its fast-growing nature and genetic similarities to MAP [22]. The host strain was cultivated in Middlebrook 7H9 media (Thermo Fisher, Mississauga, ON, Canada), incubated at 37 °C for 48 h. All 7H9 media were supplemented with 10% Oleic Albumin Dextrose Catalase growth supplement (OADC) (Thermo Fisher), 2 μg/mL mycobactin J (Allied Monitors, Montreal, QC, Canada), and 0.2% glycerol. *Mycobacterium avium* subsp. *paratuberculosis* A157 (Accession: SAMN31243806) was used in conjunction with *Mycobacterium smegmatis* mc^2^155 (Accession: NC_008596) as enrichment strains for phage isolation. This strain was streaked onto 7H11 agar plates and incubated at 37 °C in zip lock bags for 49 days. All 7H11 agar plates were supplemented with 10% OADC and 2 μg/mL mycobactin J. After 49 days, single colonies were picked using a sterile inoculation loop and transferred to Middlebrook 7H9 supplemented with 10% OADC, 2 μg/mL mycobactin J, and 0.2% glycerol, then incubated in a shaking incubator at 37 °C for 4 weeks. All mbps were plated using the double agar method [22] with 3 mL LB agar (1 mM CaCl_2_), tempered at 50 °C, and poured over 7H11 agar plates, unless stated otherwise.

### 2.2. Optimisation of a Mycobacteriophage Isolation Protocol

The optimisation protocol was developed using the *M. smegmatis* phage D29, a component of the Actiphage kit (PBD Biotech, Ipswich, UK). Firstly, a sensitivity test of the extraction method suggested by the Hatful Lab [23] was conducted. A 10-fold serial dilution of D29 at a starting concentration of 3.5 × 10^10^ PFU mL^−1^ was added to 5 g of fecal material collected from JD negative farms and homogenised. After the addition of buffer solution (10 mM Tris-HCL pH 8.0, 5 M NaCl, 1 M MgCl_2_, 1 M CaCl_2_), the samples were centrifuged and filtered using a 0.45 μm Millipore syringe filter. The filtered supernatant (100 μL) was added to 400 μL of *M. smegmatis* (OD_600_ = 1.0) and plated using the double agar method. After 24–48 h of incubation, areas of lysis were observed and counted. To optimise the sensitivity of phage isolation, 10 different treatments were considered. These treatments were chosen for their assumed ability to increase the elution of mbps from feces. These included chloroform, Tween 20, 40, and 80, Triton X, calcium chloride (CaCl_2_), sodium chloride (NaCl), glucose, EDTA, and urea. To test the effects of these treatments, we spiked 5 g of feces with 100 μL of D29 (3.5 × 10^4^ PFU mL^−1^) in buffer solution (20 mL) containing the treatments; the final concentrations can be seen in Supplementary Table S1. The diluted spiked feces aliquots were then incubated at room temperature on an analog rocking platform shaker (VWR, Mississauga, ON) for 1 h at full speed, then centrifuged at 11,000× *g* for 10 min. The supernatant was then filtered using a 0.45 μm Millipore filter and 100 μL was added to 400 μL *M. smegmatis* (OD_600_ = 1.0). After 1 h incubation at 37 °C, the samples were plated using the double agar method. Areas of lysis were observed and plaque forming units (PFUs) were counted after 24 h of incubation at 37 °C. The treatments that displayed at least a five-fold increase in the number of eluted mbps were selected for further investigation and a total of five repeats of the protocol were carried out alongside negative control of non-spiked feces. An ANOVA was conducted to see if there were any statistical differences between the treatment and standard methods. To see the effect of enrichment on our optimised method, spiked samples were incubated with 500 μL of *M. smegmatis* (OD_600_ = 1.0) and incubated overnight at 37 °C. Finally, we tested the difference between enrichment and treatment groups, by reconducting the sensitivity test with a total of four groups: no treatment, no treatment/enriched, treatment, treatment/enriched.

### 2.3. Isolation of Phages

Samples were collected from 12 dairy farms with a known presence of MAP. The MAP positivity status of these farms was obtained from an ongoing local JD control program supported by our lab group. Briefly, 5 g of environmental sample [farm soil, calf pen bedding, lactating pen feces, wastewater, pooled feces, milk sample, manure, feed, and feces direct from the cow (fresh and frozen)] was added to 10 mL of mycobacteriophage buffer (10 mM Tris-HCL pH 8.0, 5 M NaCl, 1 M MgCl_2_, 1 M CaCl_2_) and 500 μL of MAP (OD_600_ = 1.0) and 500 μL of *M. smegmatis.* Samples underwent dual enrichment in order to isolate mbps cable of lysing MAP and *M. smegmatis*. Samples were then incubated for 1–28 days. Enrichment was carried out until the isolation of a positive phage from the sample or for a maximum duration of 28 days; whichever came first. Following this enrichment procedure, 400 mM of NaCl, 0.5 mM EDTA and 0.01% Tween 80 was added to the samples. Aliquots were then placed on an analog rocking platform shaker (VWR) at full speed for up to 4 h to allow release of mbps. After time to settle (1 min), supernatant (2 mL) was removed and placed in a microfuge tube and centrifuged at room temperature at 11,000× *g* for 20 min to pellet cellular debris. This supernatant was then filter-sterilised using a 0.2 μm filter. Filter-sterilised supernatant (100 μL) was added to 400 μL of *M. smegmatis* (OD_600_ = 1.0) in a culture tube and incubated at 37 °C for 30 min. The suspension was then plated using the double agar method. Plates were incubated at 37 °C for 48 h and then examined for plaques.

### 2.4. Phage Purification and Amplification

Presumptive phage plaques were confirmed via plaque isolation and spot testing on *M. smegmatis*. Briefly, a single isolated plaque was aseptically picked from an agar plate using a glass pipette, added to 1 mL of mycobacteriophage buffer and vortexed. A total of 10 μL of this solution was spotted onto a 7H11 agar plates with a confluent lawn of *M. smegmatis* already present and incubated at 37 °C overnight. Once lysis activity was confirmed, a 10-fold serial dilution was made and 100 μL of each dilution was added to 400 μL of *M. smegmatis* (OD_600_ = 1.2). High titer phage suspensions were obtained by adding 7 mL of mycobacteriophage buffer to the plates that showed confluent lysis. After incubating overnight at 4 °C, the buffer was then recovered from the plate and filtered using a 0.2 μm filter. Purified high titer phage solutions were stored at 4 °C. Chloroform (2 μL/mL of lysate) was added to prevent a reduction in the phage titer.

### 2.5. Phage DNA Preparation

DNA was extracted using a Phage DNA Extraction kit (Norgen Biotek Corp, Thorold, ON, Canada) according to the manufacturer’s instructions. However, we used a starting volume of 2 mL of lysate (Conc. 10^8^ PFU mL^−1^) instead of the suggested 1 mL. Briefly, 13 µL of enzyme working stock solution (DNase 110 mg/mL, RNaseA 30 mg/mL, 10 × DNase1 Reaction Buffer, Nuclease free water) was added to 1300 µL of filtered phage stock. After 15 min of incubation at room temperature 1 µL of 50 mM EDTA was added, and tubes were inverted. Lysis buffer (500 µL) was added, and samples were vortexed vigorously, and then 4 µL of Proteinase K (20 mg/mL) was added. After two 15 min incubations at 55 °C and 65 °C, 320 µL of isopropanol (70%) was added to precipitate the DNA.

The lysate (2 mL) was added to spin columns and centrifuged for 1 min at 6000× *g*, this was repeated until the entire lysate volume had passed through the column. Before eluting the DNA, the spin column was washed using 400 µL of Wash Solution A, followed by centrifugation for 1 min at 6000× *g* and the flowthrough was discarded. After three washes, the column was dried thoroughly through centrifugation for 2 min at 14,000× *g*. DNA was then eluted using 75 µL of Elution Buffer B and centrifuging for 1 min at 6000× *g*. Quality and quantity of DNA was checked using a NanoVue plus spectrophotometer (GE Healthcare Life Sciences, Mississauga, ON, Canada) and the Qubit 2.0 fluorometer (Invitrogen, Burlington, ON, Canada).

### 2.6. Restriction Enzyme Digest

The restriction enzyme digests were performed with the enzymes EcoRI, AccI, NdeI, HindIII, SspI, EcoRV, PstI, and DraI (New England Biolabs, Whitby, ON, Canada). Reactions were performed per manufacturer’s instructions with a final reaction volume of 25 μL. Reactions were incubated at 37 °C for 16 h. Gel electrophoresis was then performed with the entire reaction volume on 1% agarose gel containing 0.06 μL/mL of Sybr green, using a 1 kbp ladder and run for 2 h at 80 V. Gel electrophoresis was also performed under the same conditions on non-digested phage DNA as a control.

To perform in silico digests, whole genome sequences of the phages were acquired. For this purpose, each phage genomic DNA sample was diluted to a final concentration of 0.2 ng/μL. Sequencing of these samples was performed using the Illumina MiSeq platform (Illumina, San Diego, CA, USA). Tapestation (Agilent Technologies, Santa Clara, CA, USA) was used to estimate the quantity of each library for pooling. DNA libraries for sequencing were prepared using a Nextera XT DNA library preparation kit (Illumina, San Diego, CA, USA). All sequencing steps, including cluster generation, paired-end sequencing (2 × 300 bp), and primary data analysis for quality control, were performed on the instrument. Sequence reads obtained from the MiSeq platform were checked for poorly sequenced regions and Illumina adapters sequences were trimmed using Trimmomatic [24]. After trimming sequences, filtered reads were assembled into contigs using the de novo assembly program Unicycler version 0.5.0 [25], employing built-in error correction and default parameters. Phage genome annotations and gene predictions were performed with Pharokka 1.13 [26].

### 2.7. Phylogenetic and Comparitive Genomic Analysis

After genome assembly, sequence similarity was determined using BLASTn searches in the NCBI database [27]. Following this, core phage proteins such as the major capsid protein were compared with known phages in the NCBI database using the BLASTn tool. Phages with homology to the nucleotide sequences of these phage proteins were selected to generate phylogenetic trees using Molecular Evolutionary Genetics Analysis software (MEGA) version 11.0 [28]. Phylogenies were reconstructed using the Neighbour-Joining method with 1000 bootstrap repeats [28,29].

### 2.8. MAP Susceptibility to the Phages

The previously described difficulties in working with MAP means the “gold standard” of host range assays, a plaque assay, could not be used, as the strains used do not form confluent lawns. Due to this, the phage host range was characterised using a qPCR protocol developed by Swift et al., 2013 [30]. This assay is based on the idea that if a phage is able to lyse the bacteria, DNA will be released and allow for the detection of the IS900 gene. Each reaction was run with positive controls of D29 and a negative control of whole MAP cells. Briefly, 10 μL of MAP cells were serially diluted 10-fold, with a starting concentration of 10^6^ cells. A 10 μL fraction of each dilution was then added to 90 μL of 10^8^ of each mbp lysate. This aliquot was incubated for 3.5 h at 37 °C. After incubation and centrifugation at 13,000× *g* for 3 min, real time qPCR was run to detect IS900 gene using the following primers: Forward primer: 5′-GGGAGGTCTGACGGACGT-3′, reverse primer: 5′-CGGCTGTCGGGAGTGAAG-3′ [31]. The PCR reaction mix contained 1× TaqMan Fast Universal PCR Master Mix (Thermo Fisher Scientific), 400 nM of each primer, 200 nM of the probe, and nuclease-free water. The total volume of the reaction was 20 μL. The qPCR cycling conditions were as follows: 50 °C for 2 min, 95 °C for 10 min, followed by 40 cycles of 95 °C for 15 s, 60 °C for 30 s and 72 °C for 30 s [31,32]. The qPCR was run on the BIO RAD CFX96 Real-Time System and C1000 Thermal Cycler. Alberta MAP strain A157 was used to determine whether our mbps could lyse MAP. Those with positive qPCR results were tested further using the same protocol and IS900 qPCR with other MAP strains; see Section 3.5 for strain details.

### 2.9. pH Range

To demonstrate the feasibility of these phages to be used in vivo, a range of pHs were selected that mimic the conditions found in the GI tract of cattle [33]. High titer lysates were prepared as mentioned earlier. A 1 in 50 dilution of each lysate was made in 5 mL of mycobacteriophage buffer adjusted to different pHs (5, 6, 7, and 8) with sodium hydroxide (NaOH) and concentrated hydrochloric acid (HCl). Each diluted lysate was incubated at 37 °C for 4 h. During this incubation period, 50 μL was removed after 1, 2, 3, and 4 h. A 10-fold serial dilution was then made from each 50 μL withdrawal and plated with 200 μL of *M. smegmatis* using the double agar method stated earlier.

### 2.10. Testing Phages for Cross Resistance

Resistant strains of *M. smegmatis* were generated using high titers (10^8^ PFU mL^−1^) of each phage strain. To do this, each phage was plated with 400 μL of *M. smegmatis* (OD_600_ = 1.0) using the double agar method and incubated for 48 h at 37 °C. After 48 h, bacterial colonies showing growth were selected using a sterile inoculation loop and transferred to a cell culture flask containing 20 mL of supplemented 7H9 medium. After 24 h of incubation at 37 °C in a shaking incubator, we conducted spot test assays with each of our individual mbps on the lawns of the resistant bacteria. Each assay was performed five times, and the average lysis capability was assigned based on the number of times a phage successfully lysed the resistant bacteria out of the five attempts. A score of 0/5 indicated that for all five attempts, mbps were unable to lyse the resistant bacterial strain, indicating consistent resistance, whereas 5/5 indicated that for all five attempts, the mbps were able to lyse the resistant strains, indicating no resistance. Lysis scores of less than three were considered unreliable for lysing resistant bacteria, while scores of three or more indicated successful lysis (Supplementary Figure S1).

Furthermore, we tested whether plating a high titer (10^8^ PFU mL^−1^) cocktail of our MAP-lysing mbps would reduce the number of grow-through colonies after 48, 72, and 96 h. The cocktail of mbps, mixed with 400 μL of *M. smegmatis* (OD_600_ = 1.0), was plated using the double agar method and incubated at 37 °C.

### 2.11. Testing the Virulence of mbps against Lysogens

To identify putative lysogens, a 10-fold serial dilution of each phage was spotted onto confluent *M. smegmatis* lawns. After a 48 h incubation at 37 °C, plaques were selected with bacterial overgrowth inside, and streaked onto 7H11 agar plates using an inoculation loop [34]. This purification step was repeated three times (Supplementary Figure S2). Once purified, single colonies were chosen for testing. To confirm lysogeny, colonies were streaked onto 7H11 plates, both with and without a confluent *M. smegmatis* lawn. These plates were further incubated for 48 h at 37 °C. Colonies capable of clearing the bacterial lawns were selected and cultured in 10 mL of 7H9 media. After 24 h of incubation (OD_600_ = 1.0), 400 μL of each lysogen was plated using the double agar method. A 10-fold serial dilution of each phage and a negative control (mycobacteriophage buffer) were spot-tested on each lysogen plate and then incubated at 37 °C for an additional 24 h. Subsequently, we examined the plates for plaque-forming units (PFUs) and recorded the lowest dilution resulting in a PFU to calculate the multiplicity of infection (MOI). The MOI for each phage–lysogen assay was determined by dividing the initial number of phages in the lowest dilution causing complete lysis by the initial number of lysogens added [34]. To calculate the initial titer of lysogens, we performed a 10-fold serial dilution of the lysogen culture (OD_600_ = 1.0) and plated it onto 7H11 plates. After 24 h of incubation at 37 °C, we counted colony-forming units (CFUs) to determine CFU mL^−1^. Lysogens were then assigned a susceptibility score based on the calculated MOI.

### 2.12. Efficacy of Phage Lysis of Mycobacteria in Milk Replacer and Colostrum

Dry milk replacer and bovine colostrum were obtained from SCCL (Saskatoon for colostrum) and reconstituted per manufacturer’s instructions. A total of 500 μL of 1 × 10^3^ CFU mL^−1^ of *M. smegmatis* and 100 μL of phage lysate (10^8^ PFU mL^−1^) were added to 4.9 mL of colostrum. The aliquots were then vortexed and incubated for 24 h at 37 °C. At 1, 2, 3, 4, 6, and 24 h, 100 μL was taken from these colostrum aliquots and 10 μL of Viricide (Virusol, FASTPlaqueTB kit, Biotec Laboratories Ltd., Ipswich, UK) was added to these aliquots to kill any remaining mbps in the solutions. Then, the aliquots were plated using the double agar method. After 24 h incubation, colonies were counted to determine changes in CFU mL^−1^.

## 3. Results

### 3.1. Optimisation of Isolation Protocol

**Figure 1:**
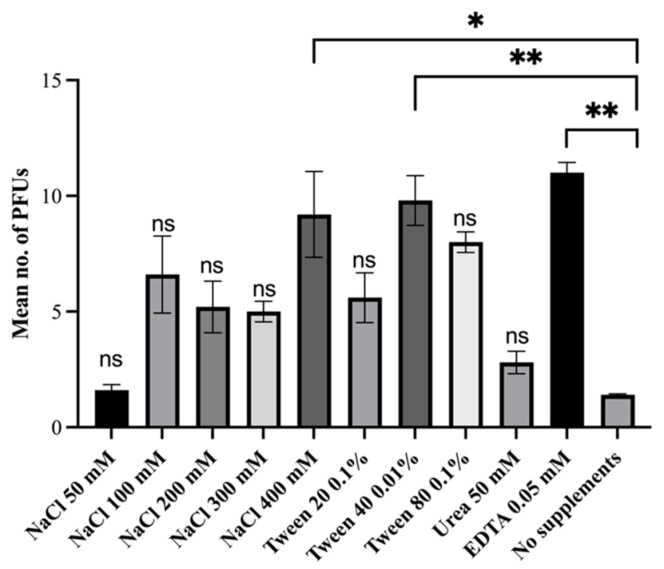
The mean plaque forming units (PFUs) eluted from spiked fecal matter as a result of the addition of chemicals. One-way ANOVA with post hoc Bonferroni test was performed between the mean PFUs eluted in the treatment group and the mean PFUs in the control group of no supplements. ‘*’ indicates a *p* value of <0.05, ‘**’ indicates a *p* value of <0.01, ‘ns’ indicates a *p* value of >0.05. Expected PFUs was 17.

To optimize the isolation protocol for the mycobacteriophages, we conducted a sensitivity test to determine the efficiency of the phage yield using our initial protocol. To calculate the phage yield, the observed number of PFUs was divided by the expected number of PFUs multiplied by 100. The expected number of PFUs was calculated via the initial titer of phages added. This sensitivity test was repeated five times and the mean number of PFUs was recorded. This test showed that the sensitivity of the original isolation protocol [23] was very low, with a phage yield from the spiked feces of less than 0.1%. We then conducted the first round of spiking feces with additions of different chemicals; the results and final concentrations are summarised in Supplementary Table S1. From these results, we further investigated the effect of EDTA, Tween 40, Tween 20, Tween 80, and sodium chloride, as they showed a five-fold increase in the number of plaques observed. From the first round of testing, we also saw a large difference between the observed PFUs and the expected number of mbps present in the sample. Due to this, we increased the elution time from 1 h to 4 h for the second round of testing. The protocol otherwise remained the same. Five repeats were performed for each treatment type alongside a negative control of the unspiked feces and untreated control. The results can be seen in Figure 1. The group treated with 400 mM sodium chloride exhibited a higher mean number of observed PFUs compared to the control group, which only received the phage spiking (*p*-value = 0.025). Similarly, there was a significant difference in the mean number of observed PFUs between the group treated with 0.1% Tween 40 and the control group (*p*-value = 0.010), as well as between the group treated with 50 mM EDTA and the control group (*p*-value = 0.001).

To further evaluate the sensitivity of this protocol, we repeated the sensitivity test with the combined treatments Tween 40, NaCl, and EDTA both with and without enrichment of mbps for 24 h. The results are shown in Table 1. The sensitivity of the isolation protocol was significantly enhanced by incorporating Tween 40, NaCl, and EDTA, resulting in an increase in yield from less than 0.01% to 59% without any enrichment. Additionally, the inclusion of Tween 40, NaCl, and EDTA elevated the sensitivity to a comparable level as enriching the original protocol. This improvement demonstrates the role of these additives in maximizing the isolation efficiency and enhancing the overall sensitivity of the protocol.

### 3.2. Isolation of Mycobacteriophages

Fourteen mycobacteriophages were isolated from the environmental samples following enrichment with MAP and *M. smegmatis* for up to 28 days. All the mbps formed plaques on the *M. smegmatis* host strain, demonstrating they are virulent in nature. The diameter of the plaques ranged from 0.1 to 3.0 mm. The phages were then purified by plaque isolation and propagation on *M. smegmatis*. For each phage, a high titer suspension (10^8^ PFU mL^−1^) was recovered from a 7H11 agar plate using 7 mL of phage buffer and stored for subsequent analysis. Table 2 shows the sources of the isolated mbps. Among the sources tested, fecal samples taken directly from dairy cows accounted for the largest proportion of the samples (237 samples). However, these samples exhibited the lowest positive sample rate, with only one mbp isolated. The next highest sample composition (118 samples) consisted of the pooled fecal samples from lactation barns, dry barns, manure pits, and outside areas. Three samples from this sample type were found to contain mbps. Interestingly, the source type with the highest success rate was feed from crops that had been fertilized with manure. Out of the 20 samples tested, four samples from two different farms yielded positive results, indicating the presence of mbps. It is worth noting that two of these mbps were identified as the same mbp. Overall, a total of eight different sample types were tested from 12 Alberta dairy farms, and mbps were successfully recovered from all but the frozen feces from dairy cows.

### 3.3. Restriction Enzyme Assay

**Figure 2:**
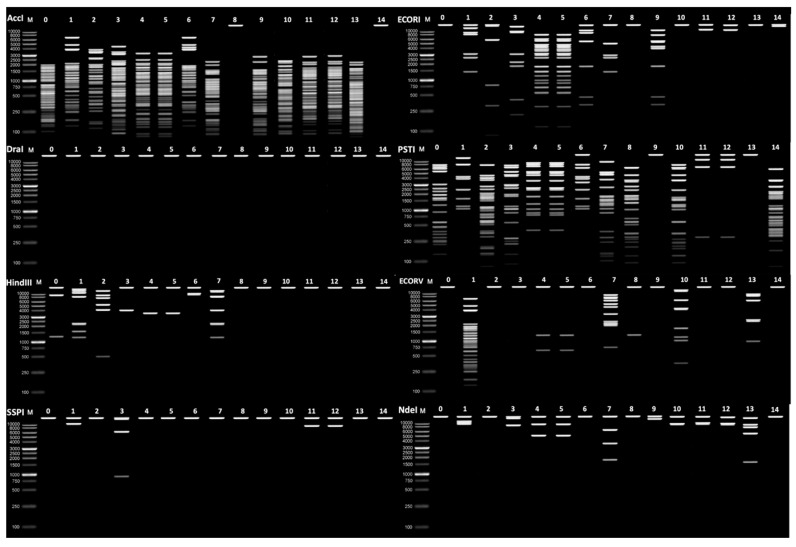
In silico restriction enzyme digest of phages isolated with *M. smegmatis* with 8 different restriction enzymes (AccI, EcoRI, DraI, PstI, HindIII, EcoRV, SspI, NdeI). 0 denotes known mycobacteriophage D29. 1 to 14 represent the mbps isolated from samples. Lane M designates 1 kbp (NEB) ladder as the “marker”.

To confirm the diversity of the isolated mbps, a restriction enzyme assay was conducted using a panel of enzymes to digest the phage DNA and the resulting banding patterns were compared. Following DNA extractions from all fourteen mbps plus D29, a series of restriction endonucleases were selected for DNA digestion (EcoRI, AccI, NdeI, HindIII, SspI, EcoRV, PstI, and DraI) (Supplementary Figure S3). To generate clear pictures, the phages were later sequenced and in silico digests were also performed and compared to the gels. The digestions performed in the lab and in silico produced the same banding patterns for each phage, providing further evidence of distinct phages. The banding patterns from these digestions were then compared (Figure 2). AccI, PstI, and HindIII were able to successfully digest D29, creating banding patterns. The enzymes NdeI, EcoRV, EcoRI, SspI, NdeI, and Dral were unable to digest D29, thus creating no banding patterns. The enzymes AccI, PstI, SspI, EcoRI, NdeI, EcoRV and HindIII were able to digest mbps 1 to 14, creating individual banding patterns to compare. Due to this, we suggest that the use of the enzymes AccI, PstI, and EcoRI would have been sufficient to achieve differentiation of the mbps. It is worth noting that no enzyme could digest all of the mbps. A comparison of the banding patterns between the fourteen mbps suggests that all the mbps isolated are distinct from D29.

However, phages 4 and 5 share identical banding patterns, as do 11 and 12, meaning they are not distinct from each other (Figure 2). This results in a total of 12 unique mycobacteriophages, with each corresponding genome sequenced assigned Genbank accession numbers, OR464701-OR464712 (Table 2).

### 3.4. Phylogenetic and Comparative Genomic Analysis

A comparative analysis was performed on all twelve phages using the complete nucleotide sequence (BLASTn) in GenBank, specifically optimized for high-similarity sequences (Megablast). This comparison revealed a similarity range of 73–98% among the sequences, confirming the isolation of twelve distinct phages. Further analysis via BLAST identified similarities between the complete genome sequences of specific phages and known phage strains. Phage 1 exhibited a 98% similarity at 41% coverage to Mycobacteriophage MilleniumForce (accession number: NC_051683). Phage 2 showed a 97% similarity at 20% coverage to *Mycobacterium* phage A6 (accession number: AP018478), while Phage 3 shared similarities with *Mycobacterium* phage Enceladus (accession number: MZ958746.1) with 96% similarity at 19% coverage. Similarly, phage 4, phage 6, phage 7, phage 9, phage 10, phage 11, phage 13, and phage 14 displayed varying degrees of similarity and coverage to different known phages. Phage 4 showed similarity to *Mycobacterium* phage 244, accession number: NC_008194 (with 98% similarity and 27% coverage). Phage 6 showed similarity to *Mycobacterium* phage Brujita, accession number: NC_011291 (with 89% similarity and 7% coverage). Phage 7 showed similarity to *Mycobacterium* phage Isiphiwo, accession number: KX6412 (with 91% similarity and 27% coverage). Phage 9 showed similarity to *Mycobacterium* phage Island3, accession number: HM152765.1 (with 98% similarity and 4% coverage). Phage 10 showed similarity to *Mycobacterium* phage Nix22, accession number: OQ383328 (with 96% similarity and 56% coverage). Phage 11 showed similarity to *Mycobacterium* phage BPs, accession number: EU568876.1 (with 98% similarity and 83% coverage). Phage 13 showed similarity to *Mycobacterium* phage Chancellor, accession number: MF140402 (with 95% similarity and 31% coverage). Phage 14 showed similarity to *Mycobacterium* phage Megabear, accession number: MH001446.1 (with 98% similarity and 76% coverage). Notably, Phage 8 exhibited a different similarity pattern to *Gordonia* phage TimTam (accession number: MH479927.1), with 75% similarity and 3% coverage. It is worth noting that all the phages except phage 10, 11, and 14 showed a query coverage less than 50%, showing that for the majority of their genomes, no homology was found to known phages, providing further evidence that the newly isolated mbps were novel. A further comparative BLAST analysis between each phage and *Mycobacterium* Phage D29 (accession number: AP018480.1) indicated significant dissimilarities for eleven of the newly isolated phages, with only phage 7 showing 81% similarity and 34% coverage.

**Figure 3:**
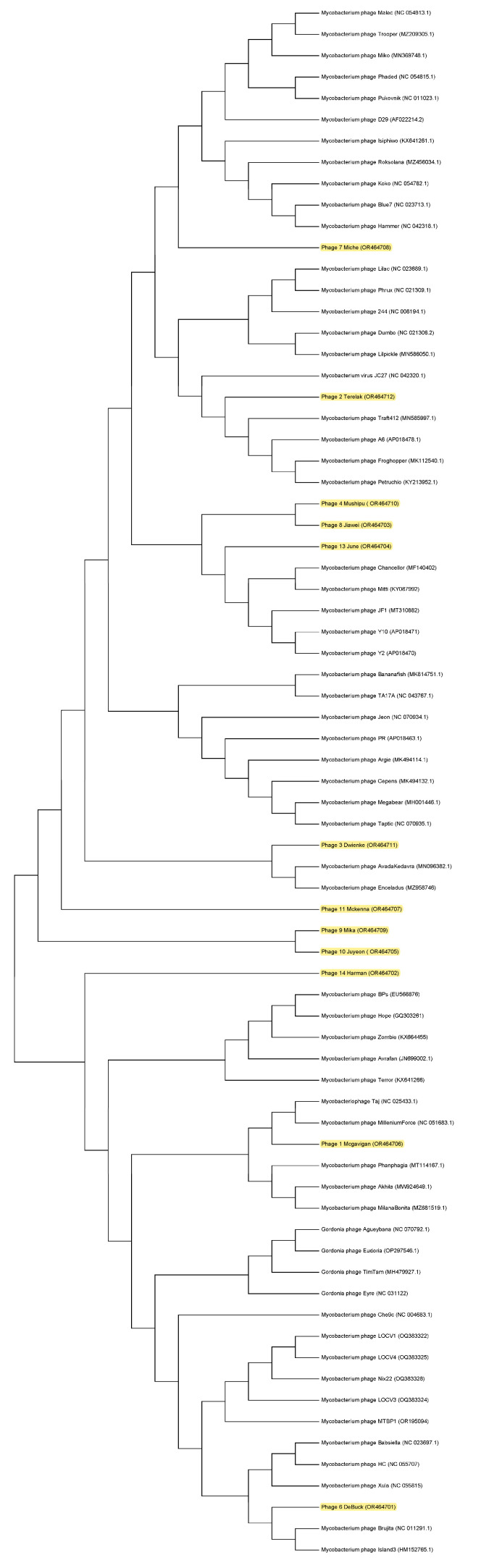
A phylogenetic tree comparing the twelve isolated phages to known phages. The phylogenetic tree was compiled with MEGA 11.0.13 software using the Maximum Likelihood method with the bootstrap test method with the number of 1000 replicates using major capsid protein sequences. The newly isolated phages are highlighted in yellow. Comparative genomic analysis revealed that twelve isolated phages are unique and novel although closely related to other Mycobacterium phages.

To construct a phylogenetic tree, predicted proteins of the large subunit of the terminase and the major capsid protein were utilized to select related phages from Genbank. Five relevant phages were chosen for each newly isolated phage, and a phylogenetic tree was constructed using MEGA 11 software (Figure 3). This comparative genomic analysis provides further evidence that 12 unique and novel phages were isolated. The twelve novel viruses were shown to be most closely related to the family Siphoviridae through phylogenetic analysis based on multiple alignments of the major phage proteins.

### 3.5. Map Susceptibility to the mbps

To assess the host range of the isolated phages, we used a qPCR protocol designed by Swift et al. [30]. This qPCR method designed by Swift et al. tests the ability of the mbps to act as a lysis agent and release bacterial DNA from low numbers of MAP cells, which can then be detected via qPCR. In this way, we firstly assessed the mbps’ ability to lyse Alberta MAP strain A157 (Accession: SAMN31243806). For each mbp, the qPCRs were run in duplicate, and the calculated average Ct values are shown in Table 2. The known phage D29 was used as a positive control, and a MAP culture that had been washed was used as a negative control. The mbps able to lyse A157 were phages 1, 2, 3, 4, 6, 7, 8, and 11. These eight phages were further tested against ten MAP strains. These ten MAP strains were selected using the phylogenetic analysis performed by Ahlstrom et al. (BioSample: SAMN03328568) [36]. We selected a representative isolate for each clade to test (Table 3). These isolates were NEIKER Bison (Accession: PRJNA565369), A1146 (Accession: SAMN03328506) A1686 (Accession: SAMN04349599), A1377 (Accession: SAMN0332856), A1154 (Accession: SAMN03328511), A1356 (Accession: SAMN03328557), and A1335 (Accession: SAMN03328551). The phages were also tested against the S type strain LN20 (EU409988.1) and C type strain K10 (PRJNA884360). For each strain of MAP, the mbps were run in duplicate and the lowest Ct score was recorded; the Ct threshold was set at 35. The results can be seen in Table 3. It is worth noting that D29 could only be used as a positive control against strain A157, as the host range of this mbp has not been fully characterised to date. The mbp that was the most successful was phage 2, as it successfully lysed eight out of ten MAP strains. The least successful mbp was phage 3, as it could only lyse two out of the ten MAP strains. Based on our host range data, to effectively lyse all 10 MAP strains, a minimum set of three mycobacteriophages is required: D29, phage 2, and either phage 8 or phage 11. In order to reduce the risk of resistance, the smallest set of mycobacteriophages needed to lyse each strain using at least two different phages should consist of six phages: D29, phage 1, phage 2, phage 7, phage 8, and phage 11. It is important to note that strains A1_335 and A1_154 are exceptions to this pattern, as they were susceptible to lysis by only one phage.

### 3.6. pH Range

**Figure 4:**
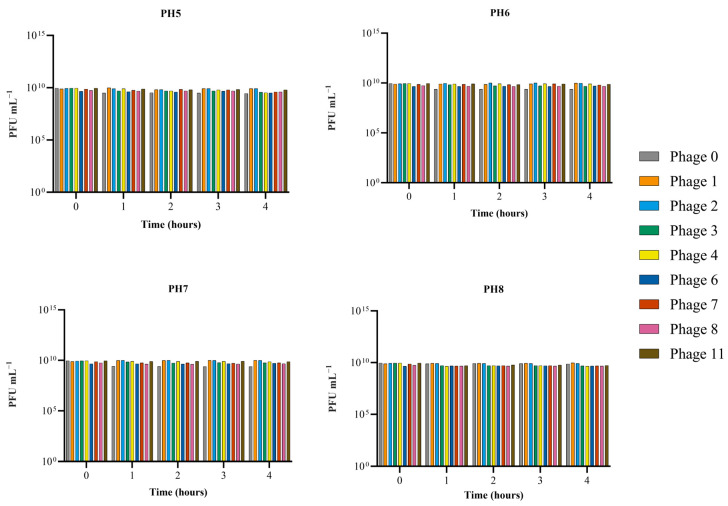
Effect of pH on mbp infectivity by exposure to varying pH for 4 h. 0 h represents the initial titer. Samples were taken at 1 h intervals and plated to assess reduction in PFU mL^−1^. Assays were performed in triplicate and phage titers were expressed as the mean.

The effect of pH was investigated for all eight phages capable of lysing MAP plus D29. After an exposure time of 1 h, all nine phages were still capable of lysing bacteria between pH 5.0 and 8.0 and showed no significant reduction in the titer (*p*-value > 0.05) after 4 h. Phage 0 showed the highest susceptibility to a change in pH (Figure 4).

### 3.7. Testing mbps for Cross Resistance

**Figure 5:**
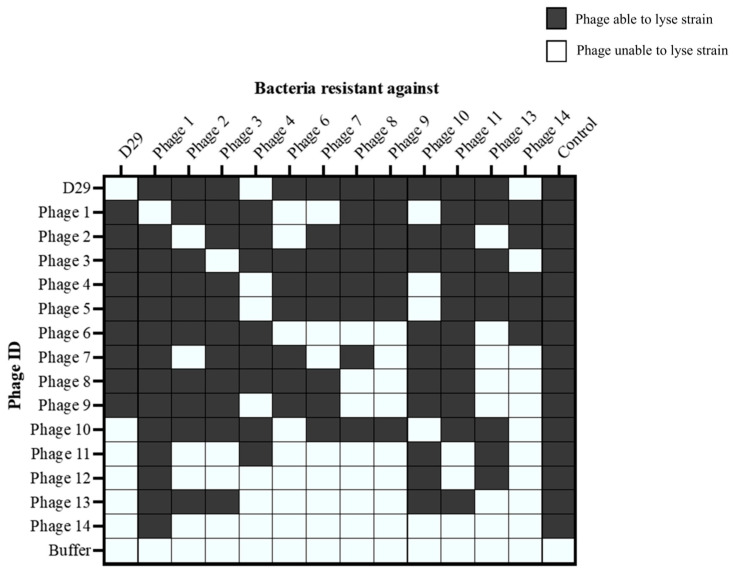
Lysis capability of mbps (0–14) spot tested on phage-resistant strains of *M. smegmatis*. The data represents the lysis capability of each phage, based on spot tests. The Y axis shows the phage tested, and the X axis shows the resistance strains of *M. smegmatis* that the phage was plated onto. The phage ID on the X axis pertains to the phage that the *M. smegmatis* strain was originally made resistant to. The lysis capability refers to the ability of the phages to form a plaque on a bacterial lawn. The control strain was *M. smegmatis* mc^2^155. Grey indicates mbp was able to lyse strain.

Spot testing assays were used to assess the resistance of bacterial strains to multiple mbps. Cross-resistance data for a panel of phage-resistant bacterial strains were visualized in a heat map (Figure 5), where the colour represents the lysis ability of each phage against each resistant bacterial strain. Overall, the results indicate that cross-resistance does not occur universally across all the mbps. The bacterial strains exhibited varying levels of resistance to different phages, suggesting that the resistance mechanisms could be specific to each phage. Phage 14 had the most resistance recorded against it, being unable to lyse 12 out of the 13 bacterial strains tested. In contrast, phage 3 and phage 4 had the least resistance recorded against them, being unable to lyse only 2 out of the 13 resistant bacterial strains. Out of those phages able to lyse MAP, the occurrence of cross-resistance was lower, at 31.53%, compared to 58.97% in the phages unable to lyse MAP. Furthermore, when a high titer cocktail of phages was plated, no grow-through colonies occurred after 96 h.

### 3.8. Testing the Virulence of mbps against Induced Lysogens

**Figure 6:**
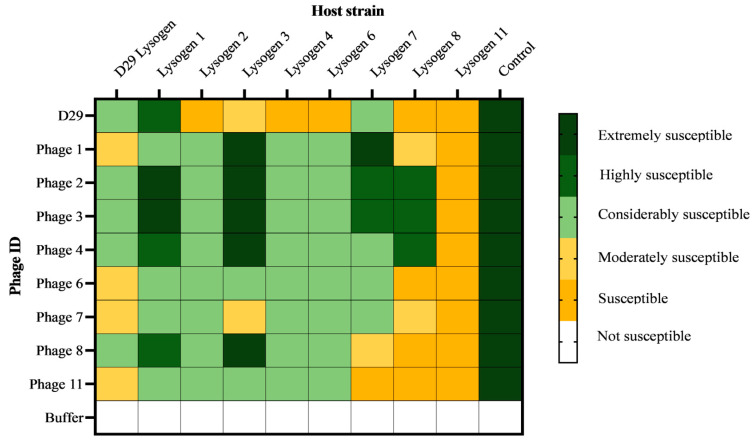
The susceptibility of lysogens to newly isolated mbps. Lysogens were assigned a susceptibility score based on the MOI. The MOI for each phage–lysogen assay was calculated by dividing the initial number of phages in the lowest dilution causing complete lysis. Those lysogens with MOI of less than 0.001, were considered extremely susceptible, 0.001 < MOI > 0.01 were highly susceptible, 0.01 < MOI > 0.1 considerably susceptible, 0.1 < MOI > 1 moderately susceptible, 1 < MOI > 10. Those lysogens with an MOI greater than 10 were considered not susceptible.

The MOI for each phage–lysogen assay was calculated by dividing the number of phages present in the highest dilution that caused plaque formation by the initial number of lysogens (bacterial cells containing prophages) added. A lower MOI suggests more efficient infection and replication of mbps within the host cells, which in turn suggests that the lysogens are more susceptible to mbps. All the phages had the ability to infect every lysogen strain. This contrasts with the resistance data, suggesting that prophages are not responsible for the resistance observed in Section 3.6. Overall, phage 1 was the most virulent against the lysogen strains, as all lysogen strains were highly susceptible to this phage with MOI values between 0.01 and 0.1. Additionally, four of the lysogen strains were extremely susceptible to phage 1, with MOI values below 0.01, indicating that phage 1 was extremely virulent against those strains. Phage 11 was the least virulent against the lysogens, as all the lysogens were only susceptible to this phage with MOI values between 1 and 10. The virulence information for the other strains can be found in Figure 6.

### 3.9. Efficacy of Phage Lysis of Mycobacteria in Milk Replacer and Colostrum

**Figure 7:**
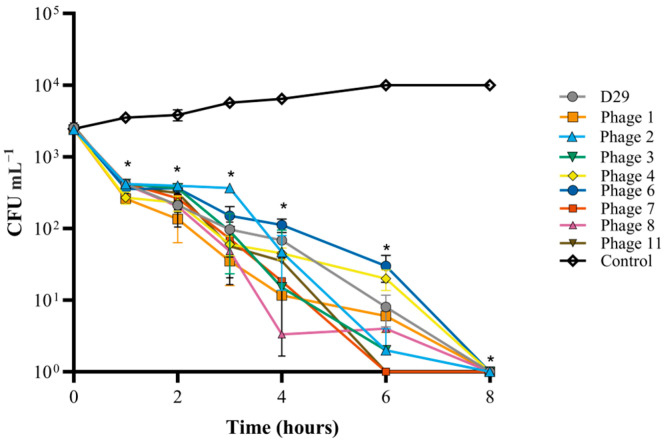
Lysis of *M. smegmatis* in reconstituted powdered colostrum spiked with mbps at 1 × 10^8^ PFU mL^−1^. Lysis was measured by reduction in CFU mL^−1^. For each phage, this was tested in quintuplicate and bacterial counts were reported as mean CFU mL^−1^ ± standard deviation. “*” indicates a significant difference compared to negative control.

**Figure 8:**
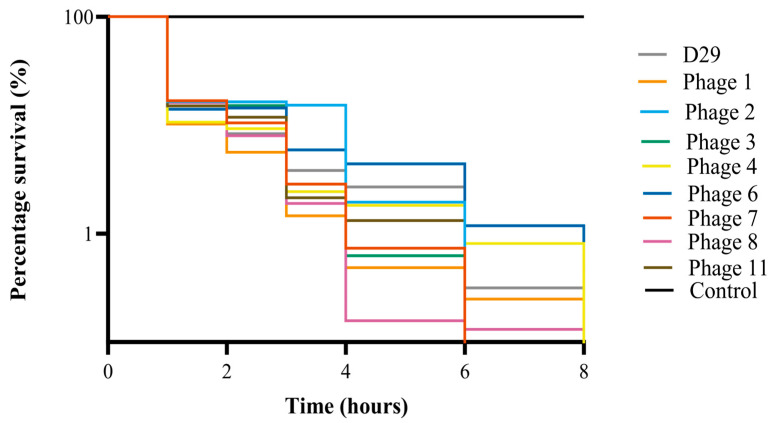
Survival curves of *M. smegmatis* in reconstituted milk replacer spiked with mbps. Bacterial cells were exposed to mbps for 12 h. The *y*-axis represents the percentage of surviving cells, and the *x*-axis represents the time in hours.

Eight mbps plus D29 were individually tested to assess their efficacy in milk replacer and colostrum. Low titers of bacteria were used to simulate the concentrations expected from environmental contamination (~1 × 10^3^ CFU mL^−1^) [37]. Reconstituted colostrum aliquots spiked with *M. smegmatis* were inoculated with high titers of each phage (1 × 10^8^ PFU mL^−1^). A Wilcoxon test was used to test the significance of the reduction in bacterial counts. For each mbp, the complete killing of *M. smegmatis* occurred within 8 h (Figure 7). When comparing the CFU mL^−1^ of *M. smegmatis* at 0 h and 8 h for each phage, there was a significant reduction in the colonies of bacteria recovered (*p* value < 0.01). Each individual phage was also tested in reconstituted milk replacer and the % survival was calculated (Figure 8). When compared to the negative control, the survival percentage was reduced to 0% within 8 h, as no CFUs could be recovered.

## 4. Discussion

In this study, twelve novel mycobacteriophages were isolated using an optimised protocol and their potential use in the control of JD was evaluated. Twelve distinct mbps were isolated from a range of environmental samples taken from MAP-positive Alberta dairy farms in 2021. The distinct banding patterns acquired by restriction enzyme analysis of mbp DNA, as well as phylogenetic and comparative analysis, indicated that these twelve mbps are distinct from each other. All mbps isolated were able to form clear plaques on *M. smegmatis* and further analysis of their host ranges revealed that eight of these mbps are able to lyse MAP. Further analysis showed the mbps’ viability and functionality in a range of pHs and in milk replacer.

Working with MAP has traditionally been challenging due to its slow-growing nature and propensity to clump [15]. These challenges have made the use of plaque assays for many strains of MAP impossible, which has resulted in a lack of mbps that are effective in killing a broad range of MAP strains and, as such, is a deficiency in our efforts to control MAP infections.

Consequently, there is a need for optimized protocols to facilitate the discovery of these phages. We have demonstrated an optimized isolation protocol by fecal spiking and the testing of different isolation solution compositions. Using this protocol, we were able to increase the phage yield from fecal samples from less than 1% to 59%. With this method, we have screened environmental samples for mbps after in-sample enrichment with MAP and the fast-growing *M. smegmatis*.

Eight MAP-lysing mbps were isolated from environmental samples taken from farms with a known presence of MAP. This is a significant achievement, given that, to date, the isolation of bacteriophages with the ability to lyse MAP has proven difficult with low yield, even after enrichment. A previous study by Basra et al. found that out of 400 samples, they were able to isolate only one mbp with the ability to lyse MAP [20]. This mbp was isolated from fecal samples from a suspected JD-positive cow. This underscores the importance of isolating phages in a location with a confirmed presence of the intended host organism, as we have demonstrated by sampling on MAP-positive farms.

Furthermore, we were able to demonstrate the susceptibility of multiple genetically disparate MAP strains to the newly isolated phages. In the past, the slow growth of MAP has made it difficult to characterize the host range of MAP-lysing mbps using the plaque assay method. Our analysis of the mbps’ host range by qPCR has revealed that each MAP strain tested could be lysed by at least one newly isolated mbp. On average, each newly isolated MAP-lysing mbp was found to be capable of lysing five out of the ten tested MAP strains. Traditionally, bacteriophages have been used for many molecular and diagnostic uses. The most common use is phage typing for diagnostic purposes. Utilising phages in diagnostics has many benefits such as specificity, cost, and time efficiency [30]. However, through our host range analysis, we have demonstrated a significant limitation of using a single phage for diagnostic tests. D29, a phage previously isolated against *M. smegmatis* from soil, has been used extensively in MAP phage assays due to its broad host range including *M. tuberculosis*, *M*. *bovis*, *M*. *avium*, *M*. *scrofulaceum*, and *M*. *ulcerans*. Most recently, in the context of MAP, a commercial product, Actiphage^®^, has been utilized for the diagnosis of JD [38]. However, from our analysis, D29 was found to be capable of lysing only five out of the ten host strains tested under specified conditions. This outcome suggests that such diagnostic tests have the potential to produce false negatives if a particular strain is unable to be lysed by the mbp used in the test. Furthermore, none of the newly isolated phages were able to lyse every strain tested, which further highlights the need for the utilisation of a cocktail of phages.

According to our findings, the mbps we have isolated have demonstrated antimycobacterial properties when spiked in colostrum. This is relevant because the ingestion of contaminated colostrum from infected dams can lead to infection [3,39]. Groenendaal et al. conducted a study which revealed that feeding calves from a bulk milk tank in which 20% of the contributing animals were infectious led to a significantly high infection rate among the calves [40]. Specifically, their findings indicate that up to 95% of youngstock may become infected through this method [39,40]. Adding phages that have the ability to lyse MAP in the colostrum would reduce the amount of viable MAP that would be ingested by the calf. Each novel mbp was able to reduce the levels of mycobacteria similar to those shown in MAP-contaminated colostrum (~1 × 10^3^ CFU mL^−1^) [37] to zero within 8 h. In addition to this, each newly isolated MAP-lysing mbp remained viable in a range of pHs, with no significant reduction in the titer observed. Colostrum is around pH 6.4 [33], suggesting that these mbps would be able to persist in colostrum for extended periods of time. Combined, this demonstrates that these novel mbps are good candidates for controlling the levels of MAP in pooled colostrum.

The newly isolated MAP-lysing mbps also showed promising results as a cocktail and individually within spiked reconstituted milk replacer, reducing mycobacterial counts to 0 within 8 h. It is worth noting that these studies were performed with the faster-growing *M. smegmatis*, as, currently, the use of MAP in such an investigation would not be possible. Not only would the slow growth rate of MAP cause difficulties but previous studies on the enumeration of MAP have shown that, due to the propensity of MAP to clump [15], CFU counts on solid media are not accurate [41]. Therefore, Optical Density must be used; however, the turbidity of colostrum and milk replacer rules out this method. These mbp cocktails have a wide range of potential uses beyond reducing infection in livestock. Phage cocktails in food safety are well-documented to prevent contamination from pathogenic bacteria. The FDA has approved ListShield™, Listex P^−1^00™, and EcoShield™ for the use of controlling *E. coli* in a range of foods such as meats, fruits, and vegetables [42,43]. MAP infections are not only related to animal welfare and economics but also potentially pose a public health risk [7]. This is due to the fact that the bacterium is hardy and thermally resistant, allowing it to survive the pasteurization of milk [8,38]. This can pose a risk, particularly to immunocompromised patients [7]. To address this issue, Endersen et al. proposed the use of a mbp cocktail before pasteurisation to ensure that there is no viable MAP left in the milk post-pasteurisation; however, they were unable to isolate MAP-lysing mbps [44]. The newly isolated mbps would be good candidates for this, as they show the ability to lyse MAP and antimycobacterial properties in reconstituted milk replacer. However, it is important to note that the antimycobacterial properties of the newly isolated mbps were investigated using the faster-growing bacterium *M. smegmatis*, and further research is needed to determine their ability to reduce MAP numbers in milk replacer and colostrum. We have demonstrated that, although 31.5% of the isolated MAP-lysing mbps were able to elicit cross-resistance against other phages, the efficacy of our cocktail to lyse mycobacterial cells was not compromised. When proposing a phage cocktail, it is also important to consider screening for cross-resistance. This means testing whether the targeted bacterial cells are resistant to more than one phage in the cocktail. If cross-resistance is common, it reduces the effectiveness of the cocktail, as the chance of a cell resistant to one phage being killed by the next phage is diminished. Therefore, ensuring that the phages in the cocktail are not cross-resistant can increase the chances of success in eliminating the targeted bacterial cells [45]. Cross-resistance occurs when a bacterium becomes resistant to one phage and, as a result, also gains resistance to other phages that share similar receptors on the bacterial surface [45]. Therefore, screening for cross-resistance in a cocktail of phages allows for the identification of phages that are more likely to be effective against the host strain, as well as select a combination of phages that are less likely to encounter cross-resistance. This can increase the success rate of phage therapy and decrease the chances of bacterial strains developing resistance. Overall, screening for resistance is a crucial step in optimizing the use of phage therapy for the treatment of bacterial infections. We found that every purposefully generated resistant strain of *M. smegmatis* was able to be lysed by the majority of the other tested phages.

In this study, we characterised both the cross-resistance of phages and lysogeny. It is important to note that these are different mechanisms by which bacteria can protect themselves from phages. No clear pattern between the occurrence of cross-resistance and the susceptibility of induced lysogens was observed, suggesting that the resistance is based on a different mechanism than the one underlying the observed lysogeny. Phage resistance refers to the ability of bacteria to resist or evade phage infection, which can occur through various mechanisms such as the modification of the bacterial cell surface, production of extracellular enzymes that degrade phage particles, or activation of the bacterial immune system to recognize and destroy phage DNA [45]. On the other hand, lysogeny is a process by which a phage infects a bacterial cell and integrates its DNA into the host cell’s genome. This results in the formation of a lysogen, which is a bacterial cell that carries the integrated phage DNA as a prophage [46]. The prophage is usually inactive and does not produce phage particles, but it can be activated under certain conditions, such as exposure to UV light or chemicals [45,46]. The mbps with the ability to become prophages would be immune to lysis from other phages, making them unsuitable for use in phage therapy [45,47]. We chose to investigate both to see if resistance to mbps was due to lysogeny or, rather, bacterial mechanisms such as CRISPR systems, which are well-characterised within bacterial genomes [48].

While characterising the effect of lysogeny within the newly isolated mbps, we found that although colonies with the characteristics of lysogens were present, they were not immune to infection by mbps. This lack of resistance suggests that the mbps isolated do not form true lysogens, but pseudolysogens [49]. Pseudolysogeny has been defined as when a phage is able to establish a long-term, non-lytic relationship with its host while maintaining its genome as a separate episome [49,50]. This is in contrast to true lysogeny, where the phage DNA integrates into the host genome and replicates as a prophage. This pseudolysogeny has important implications for the potential use of these newly isolated mbps as a prevention method for JD, as it would suggest that these mbps would not form prophages that would lead to bacterial resistance to attacking mbps used as therapeutics or in diagnostic techniques. However, further research is needed to fully understand the nature of these pseudolysogenic relationships and their potential impact on the efficacy of phage therapy. It is also worth noting that most research into this pseudolysogeny is a result of the experimental induction of lysogeny and therefore, it is hypothesised that pseudolysogeny has a lower possibility of occurring under environmental conditions [50]. The newly isolated mbps show promising results in vitro that could make them candidates for phage therapy. We have demonstrated their antimycobacterial properties and characterised a lack of lysogeny and limited cross-resistance. However, before these phages can be considered for clinical use, their efficacy and safety need to be evaluated in vivo in an animal model. Therefore, we propose that further studies be conducted to test the newly isolated phages in an animal model of experimental MAP infection.

## 5. Conclusions

In general, this study describes the isolation and characterization of eight MAP-lysing mbps and investigates their host range, cross-resistance, and viability at pH values and in colostrum. The characteristics demonstrated make these novel mbps candidates for applications in both the diagnostics and in the biocontrol of JD such as the addition to potentially contaminated colostrum. In addition to diagnostics and biocontrol, the use of a cocktail of phages could also contribute to better control of JD by preventing infection. Administering bacteriophages to calves from birth presents a potential solution to prevent MAP infection in calves. By incorporating bacteriophages into calf feed, these phages can target and eliminate MAP in the gut, preventing the establishment of infection. This early intervention could help to break transmission cycles of MAP from dam-to-calf, as well as lower calf-to-calf transmission and thereby play a crucial role in preventing the spread of JD in cattle herds. In summary, this study highlights the discovery of novel mycobacteriophages with varying abilities to lyse distinct MAP strains, thereby demonstrating their considerable potential in the control of Johne’s disease. To maximize their efficacy, we suggest the use of a phage cocktail in preventative strategies against this disease.

## Figures and Tables

**Table 1:** Final sensitivity test showing number of observed plaques with and without the addition of selected supplements and with and without enrichment.

Dilution	No. of Plaques Observed			
	With EDTA, NaCl and Tween 40		No Supplements	
	Non-Enriched	Enriched	Non-Enriched	Enriched
1	Lysed ^a^	Lysed	13	Lysed
2	Lysed	Lysed	0	Lysed
3	Lysed	Lysed	0	Lysed
4	~1000	Lysed	0	Lysed
5	278	Lysed	0	220
6	25	Lysed	0	31
7	14	Lysed	0	3
8	7	Lysed	0	1
9	4	304	0	0

^a^ Lysed indicates complete killing of bacterial lawn so no plaques were observed.

**Table 2:** Overview of the name, ID, source, and *M. avium* subsp. *paratuberculosis* lysing capacity of D29 and 14 newly isolated mbps.

Phage Isolate	Phage ID	Sample Type	Ct Value ^a,b^	Capable of Lysing MAP	Accension No.	Genome Size	Lysogeny Genes Present
D29	0	Soil [35]	27.3	Yes	AF022214.2	49,127	NA
Mcgavigan	1	Soil	27.2	Yes	OR464706	54,472	Integrase
Terelak	2	Bedding	26.7	Yes	OR464712	51,742	Integrase, Rec Proteins
Dwieneke	3	Lactating barn	17.7	Yes	OR464711	45084	Integrase, Cas4, Repressor, Anti Repressor
Mushipu	4	Feed	27.2	Yes	OR464710	74,463	Integrase, Rec Proteins (A)
Mushipu	5	Feed	27.3	Yes			
DeBuck	6	Wastewater	23.7	Yes	OR464701	49,459	Par proteins (A and B), Cas4, Repressor
Miche	7	Pooled Feces	26.3	Yes	OR464708	52,114	Integrase, Rec proteins (T), Anti Repressor
Jiawei	8	Wastewater	38.6	Yes	OR464703	41,672	Integrase
Mika	9	Milk	37.7	No	OR464709	47,102	Integrase, Rec Proteins (E and T)
Juyeon	10	Manure Pit	37.7	No	OR464705	47,422	Integrase, Rec Proteins (E and T), Repressor (A)
McKenna	11	Feed	27.7	Yes	OR464707	41,512	Integrase, Cas4, Repressor, Anti Repressor (Cro) Integrase
McKenna	12	Feed	28.1	Yes	OR464707		
June	13	Feces	38.2	No	OR464704	57,932	Integrase, Cas4, Rec Protein, Repressor
Harman	14	Pooled Feces	NA	No	OR464702	40,9374	Rec protein (A)
Control	No Phage	NA ^b^	NA	NA	NA	NA	NA

^a^ Ct value indicates cycle threshold value of IS900 qPCR detecting the release of MAP DNA, with Ct value cut off set at 35, meaning Ct value < 35 indicated an ability to lyse MAP. Duplicates for each phage were run and the lowest Ct value was used. ^b^ NA: not applicable.

**Table 3:** Host range of mbps determined by detecting MAP DNA release by IS900 qPCR.

Phage ID	MAP Strain																	
	A1_146		A1_686 (Bison)		A1_377		K10		LN20 (S Type)		NEIKER (Bison)		A1_154		A1_356		A1_335	
0	NA	No ^a^	NA	no	NA	no	23.03	yes	13.69	yes	23.9	yes	NA	no	NA	no	29.7	yes
1	34.46	yes	30.04	yes	NA	no	NA	no	35.39	no	32.24	yes	NA	no	10.89	yes	NA	no
2	29.49	yes	29.22	yes	39.5	no	23.04	yes	14.53	yes	30.05	yes	13.76	yes	14.86	yes	NA	no
3	NA	no	12.08	yes	NA	no	NA	no	35.66	no	NA	no	NA	no	NA	no	NA	no
4	26.04	yes	11.08	yes	NA	no	NA	no	33.41	yes	31.66	yes	NA	no	NA	no	NA	no
6	39.02	no	28.59	yes	NA	no	NA	no	20.49	yes	NA	no	NA	no	NA	no	NA	no
7	30.22	yes	29.22	yes	23.04	yes	NA	no	NA	no	30.87	yes	NA	no	NA	no	NA	no
8	28.73	yes	10.95	yes	39.13	no	NA	no	32.81	yes	31.69	yes	NA	no	NA	no	NA	no
11	15.84	yes	33.1	yes	27.05	yes	NA	no	16.89	yes	30.87	yes	NA	no	NA	no	NA	no
Control	39.4	no	NA	no	NA	no	NA	no	38.09	no	39.28	no	NA	no	NA	no	NA	no

^a^ The Ct values indicates cycle threshold value of IS900 qPCR detecting the release of MAP DNA, with Ct value cut off set at 35. Duplicates for each phage were run and the lowest Ct value was used. Ct value < 35 indicates ability to lyse the MAP strain.

## Data Availability

The genome sequences were deposited in GenBank under the accession numbers OR464701-OR464712. The raw data supporting the conclusions of this article will be made available by the authors, without undue reservation.

## Supplementary Materials

The following supporting information can be downloaded at https://www.mdpi.com/article/10.3390/v16010020/s1, Figure S1: Flow chart illustrating the protocol to produce resistant bacterial strains and test their susceptibility to mbps, Table S1: The number of plaque forming units (PFUs) eluted from phage spiked fecal matter as a result of the addition of chemicals compared with number of expected PFUs, Figure S2: Flow chart illustrating the protocol to produce lysogens and test their susceptibility to mbps; Figure S3: Restriction enzyme digest of phages isolated with *M. smegmatis* with 8 different enzymes (AccI, EcoRI, DraI, PstI, HindIII, EcoRV, SspI, NdeI). 0 denotes known mycobacteriophage D29. 1 to 14 denote the mbps isolated from samples. Lane M designates 1 kbp (NEB) ladder as the “marker”.

## References

[1] Tiwari A., VanLeeuwen J.A., McKenna S.L., Keefe G.P., Barkema H.W. (2006). Johne’s disease in Canada Part I: Clinical symptoms, pathophysiology, diagnosis, and prevalence in dairy herds. Can. Vet. J..

[2] Whittington R.J., Windsor P.A. (2009). In utero infection of cattle with *Mycobacterium avium* subsp. *paratuberculosis*: A critical review and meta-analysis. Vet. J..

[3] McKenna S.L., Keefe G.P., Tiwari A., VanLeeuwen J., Barkema H.W. (2006). Johne’s disease in Canada part II: Disease impacts, risk factors, and control programs for dairy producers. Can. Vet. J..

[4] Garvey M. (2020). Mycobacterium Avium Paratuberculosis: A Disease Burden on the Dairy Industry. Animals.

[5] Wolf R., Clement F., Barkema H.W., Orsel K. (2014). Economic evaluation of participation in a voluntary Johne’s disease prevention and control program from a farmer’s perspective–The Alberta Johne’s Disease Initiative. J. Dairy Sci..

[6] Ott S.L., Wells S.J., Wagner B.A. (1999). Herd-level economic losses associated with Johne’s disease on US dairy operations. Prev. Vet. Med..

[7] Hermon-Taylor J. (2009). *Mycobacterium avium* subspecies *paratuberculosis*, Crohn’s disease and the Doomsday scenario. Gut Pathog..

[8] Lund B.M., Gould G.W., Rampling A.M. (2002). Pasteurization of milk and the heat resistance of *Mycobacterium avium* subsp. *paratuberculosis*: A critical review of the data. Int. J. Food Microbiol..

[9] Fecteau M.E., Whitlock R.H., Buergelt C.D., Sweeney R.W. (2010). Exposure of young dairy cattle to *Mycobacterium avium* subsp. *paratuberculosis* (MAP) through intensive grazing of contaminated pastures in a herd positive for Johne’s disease. Can. Vet. J..

[10] National Research Council (US) Committee on Diagnosis and Control of Johne’s Disease (2003). Diagnosis and Control of Johne’s Disease.

[11] Strathdee S.A., Hatfull G.F., Mutalik V.K., Schooley R.T. (2023). Phage therapy: From biological mechanisms to future directions. Cell..

[12] Loc-Carrillo C., Abedon S.T. (2011). Pros and cons of phage therapy. Bacteriophage.

[13] Dublanchet A., Fruciano E. (2008). A short history of phage therapy. Med. Mal. Infect..

[14] Düzgüneş N., Sessevmez M., Yildirim M. (2021). Bacteriophage Therapy of Bacterial Infections: The Rediscovered Frontier. Pharmaceuticals.

[15] Rees C.E., Dodd C.E. (2006). Phage for rapid detection and control of bacterial pathogens in food. Adv. Appl. Microbiol..

[16] Cristobal-Cueto P., García-Quintanilla A., Esteban J., García-Quintanilla M. (2021). Phages in Food Industry Biocontrol and Bioremediation. Antibiotics.

[17] Dedrick R.M., E Smith B., Cristinziano M., Freeman K.G., Jacobs-Sera D., Belessis Y., Brown A.W., A Cohen K., Davidson R.M., van Duin D. (2023). Phage Therapy of Mycobacterium Infections: Compassionate Use of Phages in 20 Patients with Drug-Resistant Mycobacterial Disease. Clin. Infect. Dis..

[18] Lin D.M., Koskella B., Lin H.C. (2017). Phage therapy: An alternative to antibiotics in the age of multi-drug resistance. World J. Gastrointest. Pharm..

[19] de Juan L., Alvarez J., Romero B., Bezos J., Castellanos E., Aranaz A., Mateos A., Domínguez L. (2006). Comparison of four different culture media for isolation and growth of type II and type I/III *Mycobacterium avium* subsp. *paratuberculosis* strains isolated from cattle and goats. Appl. Env. Microbiol..

[20] Basra S., Anany H., Brovko L., Kropinski A.M., Griffiths M.W. (2014). Isolation and characterization of a novel bacteriophage against *Mycobacterium avium* subspecies *paratuberculosis*. Arch. Virol..

[21] Hyman P., Abedon S.T. (2010). Bacteriophage host range and bacterial resistance. Adv. Appl. Microbiol..

[22] Clark V.L., Bavoil P.M. (2002). Bacterial Pathogenesis. Part. C, Identification, Regulation, and Function of Virulence Factors.

[23] Russell D.A., Hatfull G.F. (2016). PhagesDB: The actinobacteriophage database. Bioinformatics.

[24] Bolger A.M., Lohse M., Usadel B. (2014). Trimmomatic: A flexible trimmer for Illumina 2400 sequence data. Bioinformatics.

[25] Wick R.R., Judd L.M., Gorrie C.L., Holt K.E. (2017). Unicycler: Resolving bacterial genome 2402 assemblies from short and long sequencing reads. PLoS Comput. Biol..

[26] Bouras G., Nepal R., Houtak G., Psaltis A.J., Wormald P.J., Vreugde S. (2023). Pharokka: A fast scalable bacteriophage annotation tool. Bioinformatics.

[27] Altschul S.F., Gish W., Miller W., Myers E.W., Lipman D.J. (1990). Basic local alignment search tool. J. Mol. Biol..

[28] Tamura K., Stecher G., Kumar S. (2021). MEGA11: Molecular Evolutionary Genetics Analysis Version 11. Mol. Biol. Evol..

[29] Mohammadi M., Saffari M., Siadat S.D., Hejazi S.H., Shayestehpour M., Motallebi M., Eidi M. (2023). Isolation, characterization, therapeutic potency, and genomic analysis of a novel bacteriophage vB_KshKPC-M against carbapenemase-producing Klebsiella pneumoniae strains (CRKP) isolated from Ventilator-associated pneumoniae (VAP) infection of COVID-19 patients. Ann. Clin. Microbiol. Antimicrob..

[30] Swift B.M., Denton E.J., Mahendran S.A., Huxley J.N., Rees C.E. (2013). Development of a rapid phage-based method for the detection of viable *Mycobacterium avium* subsp. *paratuberculosis* in blood within 48 h. J. Microbiol. Methods.

[31] Bull T.J., McMinn E.J., Sidi-Boumedine K., Skull A., Durkin D., Neild P., Rhodes G., Pickup R., Hermon-Taylor J. (2003). Detection and verification of *Mycobacterium avium* subsp. *paratuberculosis* in fresh ileocolonic mucosal biopsy specimens from individuals with and without Crohn’s disease. J. Clin. Microbiol..

[32] Altamirano F.L.G., Barr J.J. (2021). Screening for Lysogen Activity in Therapeutically Relevant Bacteriophages. Bio Protoc..

[33] Puppel K., Gołębiewski M., Grodkowski G., Slósarz J., Kunowska-Slósarz M., Solarczyk P., Łukasiewicz M., Balcerak M., Przysucha T. (2019). Composition and Factors Affecting Quality of Bovine Colostrum: A Review. Animals.

[34] Storms Z.J., Teel M.R., Mercurio K., Sauvageau D. (2020). The Virulence Index: A Metric for Quantitative Analysis of Phage Virulence. Phage.

[35] Froman S., Will D.W., Bogen E. (1954). Bacteriophage active against virulent *Mycobacterium tuberculosis*. I. Isolation and activity. Am. J. Public Health Nations Health.

[36] Ahlstrom C., Barkema H.W., Stevenson K., Zadoks R.N., Biek R., Kao R., Trewby H., Haupstein D., Kelton D.F., Fecteau G. (2016). Genome-Wide Diversity and Phylogeography of *Mycobacterium avium* subsp. *paratuberculosis* in Canadian Dairy Cattle. PLoS ONE.

[37] Grant I.R., Ball H.J., Rowe M.T. (2002). Incidence of *Mycobacterium paratuberculosis* in bulk raw and commercially pasteurized cows’ milk from approved dairy processing establishments in the United Kingdom. Appl. Environ. Microbiol..

[38] Swift B.M., Meade N., Barron E.S., Bennett M., Perehenic T., Hughes V., Stevenson K., Rees C.E. (2020). The development and use of Actiphage(^®^) to detect viable mycobacteria from bovine tuberculosis and Johne’s disease-infected animals. Microb. Biotechnol..

[39] Grant I.R. (2021). Bacteriophage-Based Methods for Detection of Viable *Mycobacterium avium* subsp. *paratuberculosis* and Their Potential for Diagnosis of Johne’s Disease. Front. Vet. Sci..

[40] Groenendaal H., Nielen M., Jalvingh A.W., Horst S.H., Galligan D.T., Hesselink J.W. (2002). A simulation of Johne’s disease control. Prev. Vet. Med..

[41] Carter C.D., Parks A., Abuladze T., Li M., Woolston J., Magnone J., Senecal A., Kropinski A.M., Sulakvelidze A. (2012). Bacteriophage cocktail significantly reduces *Escherichia coli* O157: H7 contamination of lettuce and beef but does not protect against recontamination. Bacteriophage.

[42] Kralik P., Beran V., Pavlik I. (2012). Enumeration of *Mycobacterium avium* subsp. *paratuberculosis* by quantitative real-time PCR, culture on solid media and optical densitometry. BMC Res. Notes.

[43] Moye Z.D., Woolston J., Sulakvelidze A. (2018). Bacteriophage Applications for Food Production and Processing. Viruses.

[44] Endersen L., Coffey A., Neve H., McAuliffe O., Ross R.P., O’mahony J. (2013). Isolation and characterisation of six novel mycobacteriophages and investigation of their antimicrobial potential in milk. Int. Dairy J..

[45] Gill J.J., Hyman P. (2010). Phage choice, isolation, and preparation for phage therapy. Curr. Pharm. Biotechnol..

[46] Casjens S. (2003). Prophages and bacterial genomics: What have we learned so far?. Mol. Microbiol..

[47] Shao Q., Trinh J.T., McIntosh C.S., Christenson B., Balázsi G., Zeng L. (2017). Lysis-lysogeny coexistence: Prophage integration during lytic development. Microbiologyopen.

[48] Barrangou R., Horvath P. (2012). CRISPR: New horizons in phage resistance and strain identification. Annu. Rev. Food Sci. Technol..

[49] Baess I. (1971). Report on a pseudolysogenic *Mycobacterium* and a review of the literature concerning pseudolysogeny. Acta Pathol. Microbiol. Scand. B Microbiol. Immunol..

[50] Łoś M., Węgrzyn G. (2012). Pseudolysogeny. Adv. Virus Res..

