# Supplementary material for: Effective Isolation and Characterization of Mycobacteriophages with the Ability to Lyse Mycobacterium avium subsp. paratuberculosis
Source: Viruses. 2023 Dec 22;16(1):20. doi: 10.3390/v16010020 (PMC10819923; doi:10.3390/v16010020)
Supplement: Supplementary file 1 [file viruses-16-00020-s001.zip › Supplementary figures.pdf]

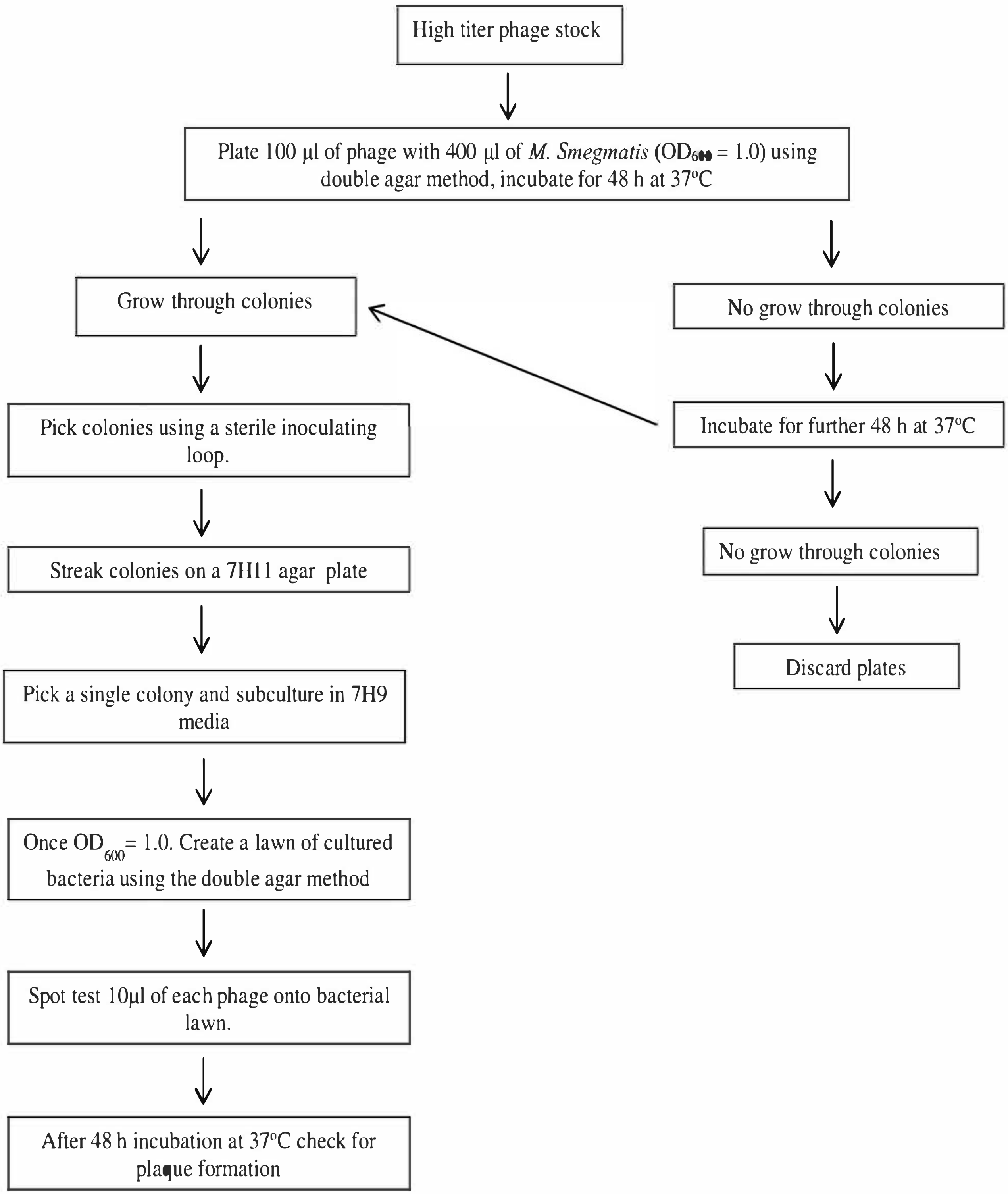

Figure S1. Flow chart illustrating the protocol to produce resistance bacterial strains and test their susceptibility to mbps

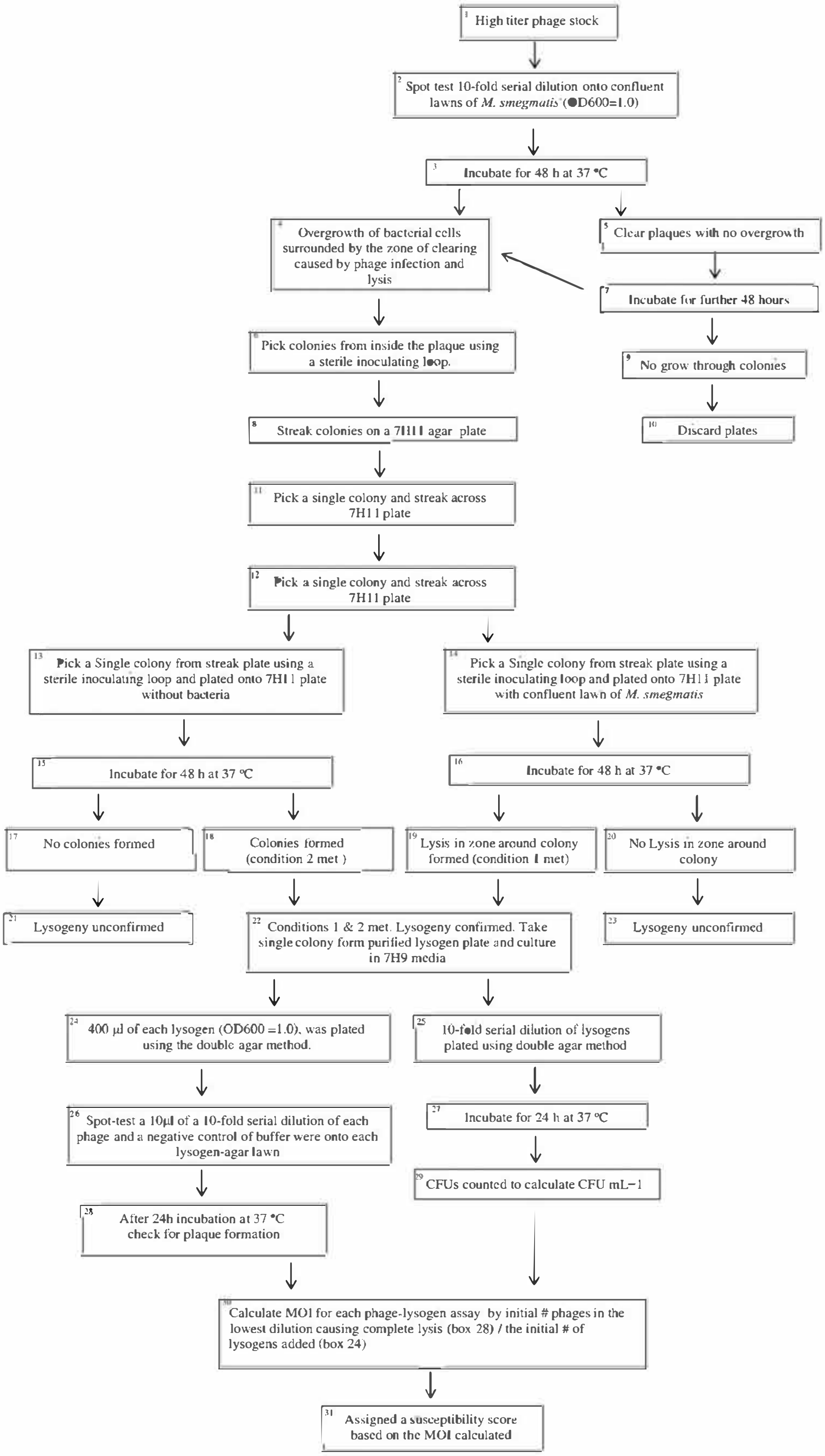

Figure S2. Flow chart illustrating the protocol to produce lysogens and test their susceptibility to mbps

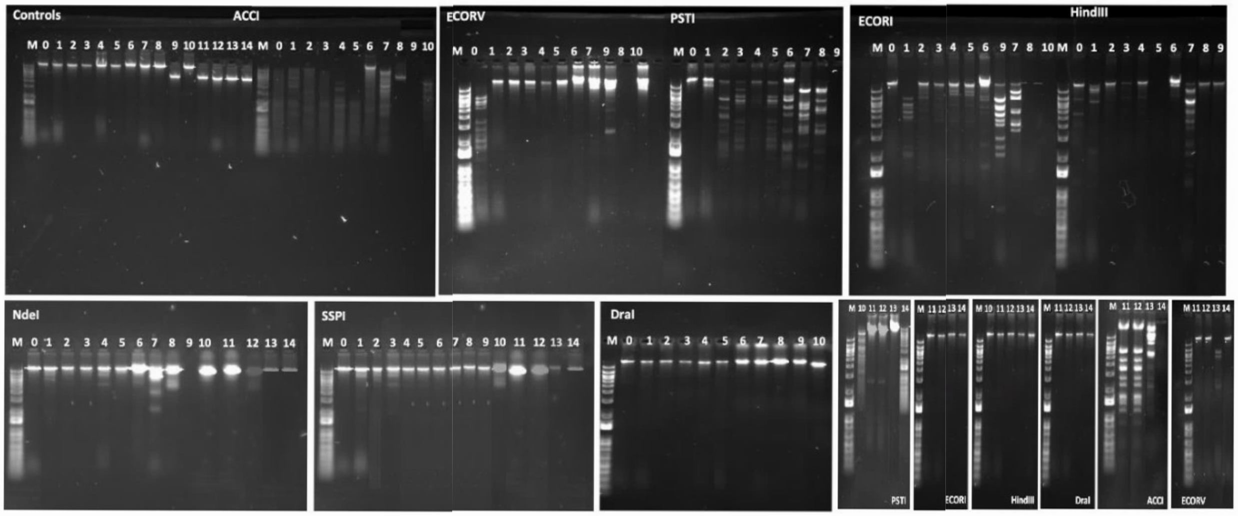

Figure S3. Restriction enzyme digest of phages isolated with *M. smegmatis* with 8 different restriction enzymes (AccI, EcoRI, DraI, PstI, HindIII, EcoRV, SspI, NdeI). 0 denotes known mycobacteriophage D29. 1 to 14 represents the mbps isolated from samples. Lane M designates 1 kbp (NEB) ladder as the “marker”.
