# Supplementary material for: Effective Isolation and Characterization of Mycobacteriophages with the Ability to Lyse Mycobacterium avium subsp. paratuberculosis
Source: Viruses. 2023 Dec 22;16(1):20. doi: 10.3390/v16010020 (PMC10819923; doi:10.3390/v16010020)
Supplement: Supplementary file 1 [file viruses-16-00020-s001.zip › Table S1.pdf]

Table S1. The number of plaque forming units (PFUs) eluted from phage spiked fecal matter as a result of the addition of chemicals compared with number of expected PFUs.

**Treatment**	**Final Concentration**	**PFUS Observed /Expected**
No supplements	NA	1/17
No Phage	NA	0/0
Chloroform	3 ul/g of sample	2/17
Tween 80	0.01 %	5/17
Tween 40	0.01 %	3/17
Tween 20	0.01 %	4/17
Triton X	0.01 %	3/17
EDTA	0.05 mM	7/17
Urea	50 mM	3/17
Calcium Chloride	100 mM	3/17
	200 mM	1/17
	300 mM	1/17
	400 mM	0/17
	500 mM	0/17
Sodium Chloride	100 mM	2/17
	200 mM	6/17
	300 mM	5/17
	400 mM	1/17
	500 mM	0/17
Glucose and Maltose mix	200 mM	0/17
	200mM	0/17
	300 mM	0/17
	400 mM	0/17
	500 mM	0/17
